# Regulation of cell growth and migration by miR-96 and miR-183 in a breast cancer model of epithelial-mesenchymal transition

**DOI:** 10.1371/journal.pone.0233187

**Published:** 2020-05-12

**Authors:** Olivia Anderson, Irene K. Guttilla Reed

**Affiliations:** Department of Biology, University of Saint Joseph, West Hartford, Connecticut, United States of America; University of Colorado Boulder, UNITED STATES

## Abstract

Breast cancer is the most commonly diagnosed malignancy in women, and has the second highest mortality rate. Over 90% of all cancer-related deaths are due to metastasis, which is the spread of malignant cells from the primary tumor to a secondary site in the body. It is hypothesized that one cause of metastasis involves epithelial-mesenchymal transition (EMT). When epithelial cells undergo EMT and transition into mesenchymal cells, they display increased levels of cell proliferation and invasion, resulting in a more aggressive phenotype. While many factors regulate EMT, microRNAs have been implicated in driving this process. MicroRNAs are short noncoding RNAs that suppress protein production, therefore loss of microRNAs may promote the overexpression of specific target proteins important for EMT. The goal of this study was to investigate the role of miR-96 and miR-183 in EMT in breast cancer. Both miR-96 and miR-183 were found to be downregulated in post-EMT breast cancer cells. When microRNA mimics were transfected into these cells, there was a significant decrease in cell viability and migration, and a shift from a mesenchymal to an epithelial morphology (mesenchymal-epithelial transition or MET). These MET-related changes may be facilitated in part by the regulation of ZEB1 and vimentin, as both of these proteins were downregulated when miR-96 and miR-183 were overexpressed in post-EMT cells. These findings indicate that the loss of miR-96 and miR-183 may help facilitate EMT and contribute to the maintenance of a mesenchymal phenotype. Understanding the role of microRNAs in regulating EMT is significant in order to not only further elucidate the pathways that facilitate metastasis, but also identify potential therapeutic options for preventing or reversing this process.

## Introduction

Breast cancer is the most commonly diagnosed malignancy in women, with approximately 1 in every 8 women at risk for the disease [1]. There are five clinical subtypes of breast cancer, which are characterized by the nature of the cells that make up the tumor [1]. The most common type of breast cancer, Luminal A, is characterized by an epithelial cell type, which typically indicates a better prognosis due to the low-level of invasiveness of the cells [2]. The characteristics of the epithelial cells found in some breast cancers include tight cell-cell junctions and cell-matrix adhesion, resulting in a cuboidal cell morphology with very low motility [2]. However, other types of breast cancer, such was Basal-like and Claudin-low, display mesenchymal cell characteristics including increased rates of cell growth, invasion, and metastasis [2]. One mechanism that promotes metastasis is the invasion of cancerous cells across the basement membrane, facilitating their entrance into the circulatory or lymphatic system [3]. This can result in the spread of the primary tumor to secondary sites in the body. The metastasis of tumors is responsible for over 90 percent of cancer-related deaths [4], therefore understanding the mechanisms that control this process is crucial to monitoring and treating cancer.

It is hypothesized that the first step in the complex metastatic process for carcinomas is epithelial-mesenchymal transition (EMT) [3]. Mesenchymal cells are characterized by their loss of cell-cell junctions and cell-matrix adhesion. Furthermore, during EMT cells undergo changes in cytoskeletal proteins such as the upregulation of vimentin and fibronectin, resulting in a spindle-shaped morphology with increased cellular motility [3]. These changes cause an increase in the invasiveness of the cancer cells. It is hypothesized that EMT is driven by specific molecular changes, including dysregulation of microRNAs [3].

MicroRNAs are small segments of noncoding RNA that regulate protein expression [5]. MicroRNAs negatively regulate gene expression by binding to target mRNAs resulting in either degradation of those mRNAs or translational inhibition [5]. Increasing or decreasing the levels of specific microRNAs can result in aberrant protein expression, leading to the initiation or progression of EMT. Previous research has shown that certain microRNAs are downregulated during EMT, suggesting that they may play a role in regulating this process [3]. The focus of this study was to identify microRNAs that are downregulated during EMT and determine if these changes impact cellular phenotype via targeting proteins that play a role in this transition. It is hypothesized that proteins targeted by downregulated microRNAs may create or maintain the aggressive mesenchymal phenotype resulting from EMT in breast cancer.

The cell lines that were used in this study were epithelial MCF-7 and mesenchymal MCF-7_M_ cells. MCF-7_M_ cells were derived from MCF-7 cells following prolonged mammosphere culture, which was previously shown to induce EMT [6]. Using these cell lines as a pre- and post-EMT model, two microRNAs (miR-96 and miR-183) were shown to be significantly downregulated in MCF-7_M_ vs. MCF-7 cells, indicating that they may play a role in EMT. The miR-183-96-182 microRNA cluster has been shown to have both oncogenic and tumor suppressive effects in different types of cancers, playing content- dependent roles in cell proliferation, migration, and metastasis [7]. However, there is conflicting evidence surrounding the role of these microRNAs in EMT in breast cancer [8–10]. This study further elucidates the role of miR-96 and miR-183 in EMT by utilizing stable pre- and post-EMT cells derived from the same parental breast cancer cell line, minimizing genetic variation. We also provide evidence that the loss of miR-96 and miR-183 helps to maintain the mesenchymal phenotype, suggesting that these microRNAs could be used clinically to induce a less aggressive phenotype resulting in a more treatable cancer.

## Materials and methods

### Cell culture

MCF-7 cells were obtained from the American Type Culture Collection (ATCC® HTB-22^™^). MCF-7_M_ cells were obtained from our collaborator Dr. Bruce White at UConn Health in Farmington, CT [6]. MCF-7 and MCF-7_M_ cells were cultured in media containing DMEM/F12, 10% fetal bovine serum, and 1% penicillin/streptomycin and passaged weekly.

### MicroRNA expression array

RNA was isolated from MCF-7 and MCF-7_M_ cells using TRIzol, and cDNA was synthesized using the RT2 microRNA First Strand Kit from l g of total RNA. Human Cancer RT2 microRNA PCR arrays were run on an Applied Biosystems 7900 HT Fast Real-time PCR system according to the manufacturer’s protocol. The temperature program used for qRT-PCR was as follows: 50°C for 2 min; 95°C for 1 min; 95°C for 30 sec and 55°C for 1 min (40 cycles); 95°C for 15 sec; 55°C for 15 sec; 95°C for 15 sec. Data was analyzed using online software from SA Biosciences available at https://dataanalysis.sabiosciences.com/pcr/arrayanalysis.php (SNORD44 was used as reference gene).

### RNA isolation

RNA was isolated from MCF-7 and MCF-7_M_ cell lines using TRIzol according to the manufacturer’s protocol. RNA quantity and purity was measured using a Nanodrop One C spectrophotometer.

### cDNA synthesis and PCR

To measure microRNA expression, cDNA was synthesized using microRNA-specific primers (S1 Table) from 2 μg of total RNA using SuperScript^™^ III Reverse Transcriptase. PCR was carried out using *Taq* polymerase,standard buffers, and microRNA-specific primers (S1 Table). The temperature program used for endpoint PCR was as follows: 95°C for 5 min; 95°C for 30 sec, 55°C for 30 sec, and 72°C for 30 (32 cycles); 72°C for 10 min. MicroRNAs amplified using PCR were resolved on a 10% non-denaturing acrylamide gel and visualized using SYBR Gold stain.

### Western blot

Whole cell lysates were prepared using RIPA buffer supplemented with fresh protease inhibitors. Protein was resolved on a 10% SDS-PAGE gel in 1X Tris/Glycine/SDS buffer, and transferred to a nitrocellulose membrane in 1X Tris/Glycine buffer with 20% methanol at 20 V overnight. Membranes were blocked overnight with 5% milk in TBST at 4°C, then incubated with α-ZEB1 (1:500) or α-β-Actin (1:1000) primary antibodies in 5% milk in TBST at 4°C overnight. ZEB1 antibodies were obtained from Cell Signaling Technology, TCF8/ZEB1 [D80D3] rabbit IgG mAb #3396; Antibody Registry public ID #AB_1904164. β-actin antibodies were obtained from Santa Cruz Biotechnology (β-Actin Antibody [C4] mouse IgG_1_ mAb #sc-47778; Antibody Registry public ID #AB_2714189). Following triplicate washes with fresh TBST, membranes were incubated with α-rabbit (ZEB1) or α-mouse (β-actin) IgG antibodies conjugated to alkaline phosphatase. Membranes were incubated with secondary antibodies at a dilution of 1:500 in 5% milk in TBST for one hour, then developed using BCIP/NBT solution. Bands from two independent transfections were quantitated using ImageJ software.

### Transfection

MCF-7_M_ and MCF-7 cells were seeded into the appropriate cell culture dish and allowed to adhere overnight. MicroRNA levels were increased in MCF-7_M_ cells by transfecting 50nM of the appropriate mirVana^™^ miRNA mimics into cells, while microRNA levels were decreased in MCF-7 cells by transfecting 50 nM of the appropriate mirVana^™^ miRNA inhibitors into cells. Mimics and inhibitors were transfected into cells displaying 80% confluency using RNAiMAX transfection reagent diluted in Opti-MEM^™^ I Reduced Serum Medium. As a negative control, 50nM of either mirVana^™^ miRNA mimic or inhibitor, Negative Control #1 was transfected into cells. Complete media was added back to the cells after 4 hours, and cells were incubated for 24 or 48 hours before assays were performed.

### Wound healing assay

MCF-7 cells were seeded at a density of 3 x 10^5^ cells per well, and MCF-7_M_ cells were seeded at a density of 1.5 x 10^5^ cells per well in a 6 well dish, and were allowed to adhere overnight. To prevent over growth, MCF-7_M_ cells were seeded at a lower density due to their higher rate of proliferation as compared to MCF-7 cells [6]. Cells were transfected with 50 nM of either mirVana^™^ miRNA inhibitors (MCF-7) or mirVana^™^ miRNA mimics (MCF-7_M_) as well as negative controls. Once the cells reached confluence, triplicate wounds were created in each well using a sterile p-10 pipette tip. Images were captured at the indicated increments following creation of the wound using the EVOS FLoid® Cell Imaging Station. Relative wound width was measured using ImageJ software, and statistical significance was determined using a one-way ANOVA followed by a Tukey-Kramer post-hoc analysis.

### Trypan blue cell viability assay

MCF-7 cells were seeded at a density of 3 x 10^5^ cells per well and MCF-7_M_ cells were seeded at a density of 1.5 x 10^5^ cells per well in a 6 well dish and were allowed to adhere overnight. To prevent over growth, MCF-7_M_ cells were seeded at a lower density due to their higher rate of proliferation as compared to MCF-7 cells [6]. Cells were then transfected with 50 nM of either mirVana^™^ miRNA inhibitors (MCF-7) or mirVana^™^ miRNA mimics (MCF-7_M_) as well as negative controls. At the indicated time points, cells were trypsinized and stained with 0.4% trypan blue solution to determine the number of viable cells. In each experiment cells were counted in triplicate at both 24 and 48 hour time points. Statistical significance was determined using a one-way ANOVA followed by a Tukey-Kramer post-hoc analysis.

### MTT assay

MTT assays were performed using the Vybrant® MTT Cell Proliferation Assay Kit. MCF-7 cells were seeded at a density of 1.4 x 10^4^ cells per well and MCF-7_M_ cells were seeded at a density 0.7 x 10^4^ cells per well in a 96-well plate and were allowed to adhere overnight. Cells were then transfected with 50 nM of either mirVana^™^ miRNA inhibitors (MCF-7) or mirVana^™^ miRNA mimics (MCF-7_M_) as well as negative controls, and incubated for 48 hours. 10 μl of MTT reagent was added to each well, and the plate was incubated for an additional 4 hours at 37°C. Then, 100 μl of SDS-HCl solution was added to each well, and the plate was incubated for 18 hours at 37°C. Absorbance at 570 nm was determined using a Varioskan LUX Multimode Microplate Reader.

### Immunofluorescence

MCF-7 cells were seeded at a density of 7 x 10^3^ cells per well and MCF-7_M_ cells were seeded at a density of 1.4 x 10^4^ cells per well in 24 well dishes and were allowed to adhere overnight. Cells were then transfected with 50nM of either mirVana^™^ miRNA inhibitors (MCF-7) or mirVana^™^ miRNA mimics (MCF-7_M_) as well as negative controls, and incubated for 48 hours. Media was removed and cells were fixed in 4% formaldehyde for 10 minutes at 37°C. Cells were then washed with PBS and permeabilized in 0.5% Triton X-100 for 15 minutes at room temperature. Cells were then washed in triplicate with PBS and incubated in blocking solution (3% BSA) for 1 hour. Blocking solution was then removed and α-vimentin primary antibodies were added (1:100 dilution in 3% BSA) and left overnight at 4°C. Vimentin antibodies were obtained from Santa Cruz Biotechnology (Vimentin Antibody [H-84] rabbit polyclonal Ab #sc-5565; Antibody Registry public ID # AB_793999). The cells were then washed with PBS and incubated in blocking solution containing 1 drop of goat anti-rabbit IgG (H+L) Cross-Adsorbed ReadyProbes^™^ Alexa Fluor 594 secondary antibody at room temperature for 30 minutes. Cells were again washed with PBS before 400μl of Live Cell Imaging Solution and 1 drop of NucBlue^™^ Fixed Cell ReadyProbes^™^ Reagent was added to each well and incubated at room temperature for 5 minutes. Cells were than imaged with a EVOS FLoid® Cell Imaging Station.

## Results

**Fig 1:**
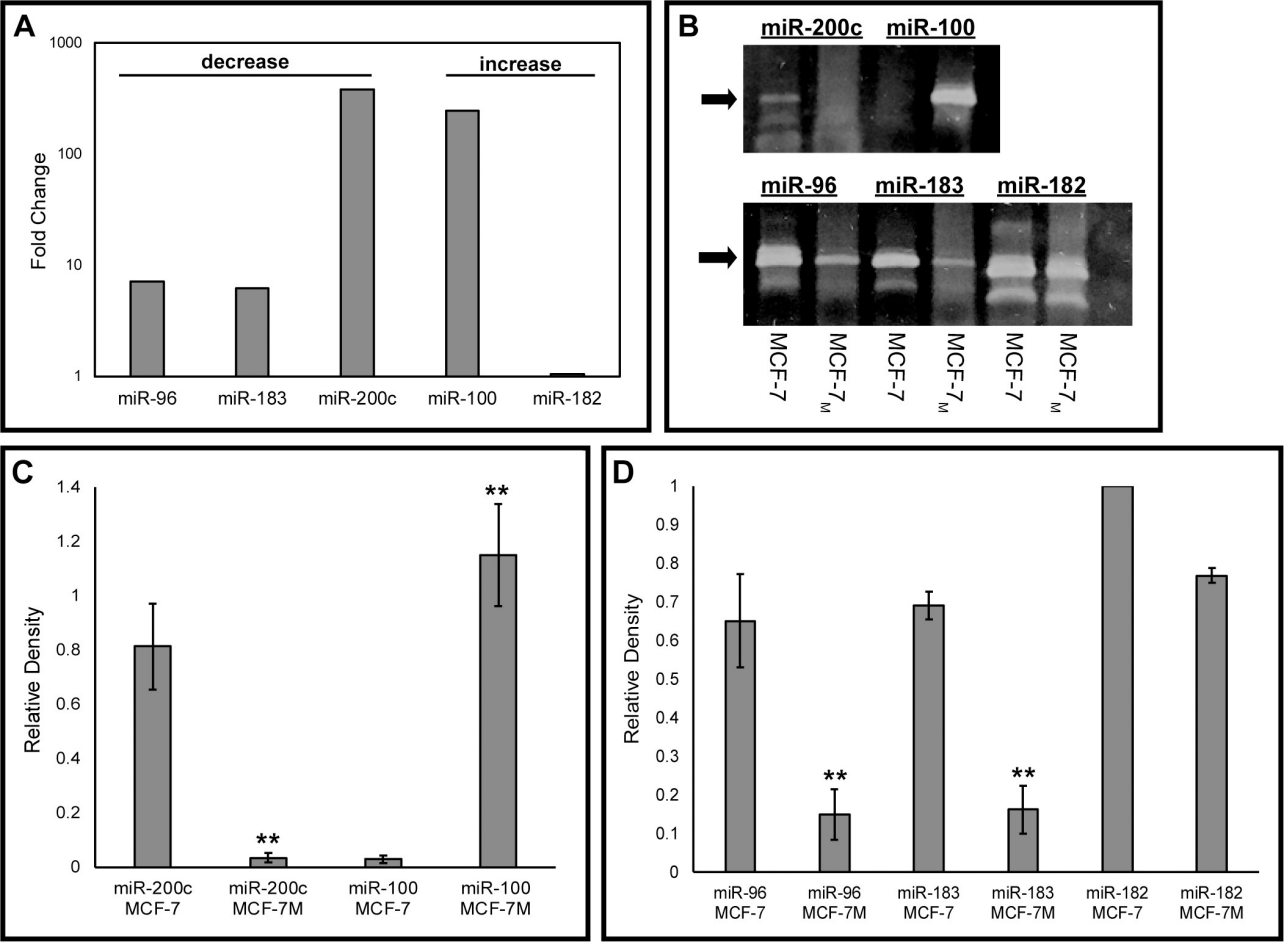
MicroRNAs miR-96 and miR-183 are downregulated in post-EMT MCF-7_M_ breast cancer cells. (A) Results from two ‘‘Cancer microRNA” RT2 microRNA PCR arrays performed on MCF-7 and MCF-7_M_ cell RNA samples. Values are presented as fold-change on a log scale (decreased, left; increased, right) in MCF-7_M_ cells compared to MCF-7 cells. (B) Endpoint PCR was used to confirm relative levels of specific microRNAs in MCF-7 and MCF-7_M_ cell lines. PCR products were resolved on a 10% non-denaturing acrylamide gel and stained with SYBR Gold. Arrow indicates expected band size (72 bp). (C, D) Relative expression levels of microRNAs (expressed as averages ± SEM) were quantitated using ImageJ software for independent experiments. Statistical significance was determined using a one-way ANOVA followed by a Tukey-Kramer post-hoc analysis. **p < .01.

This study aimed to examine the role of miR-96 and miR-183 in a breast cancer model of EMT. Therefore, first the levels of microRNAs in the miR-183-96-182 cluster were analyzed in the two cell lines of interest, MCF-7 vs. MCF-7_M_, using a PCR microarray for cancer microRNAs (Fig 1A). Levels of miR-96 and miR-183 were 6 to 7-fold lower in MCF-7_M_ cells vs. MCF-7 cells. The controls miR-200c and miR-100 had the predicted responses, as miR-200c is known to be downregulated and miR-100 is known to be upregulated in MCF-7_M_ vs. MCF-7 cells [11]. The relative expression of these microRNAs was then confirmed using semi-quantitative endpoint RT-PCR (Fig 1B–1D). Minimal differences in expression levels were observed for miR-182 in MCF-7_M_ vs. MCF-7 cells and expression levels were not consistent between the microRNA microarray and PCR validation, therefore this microRNA was not included in subsequent analyses.

**Fig 2:**
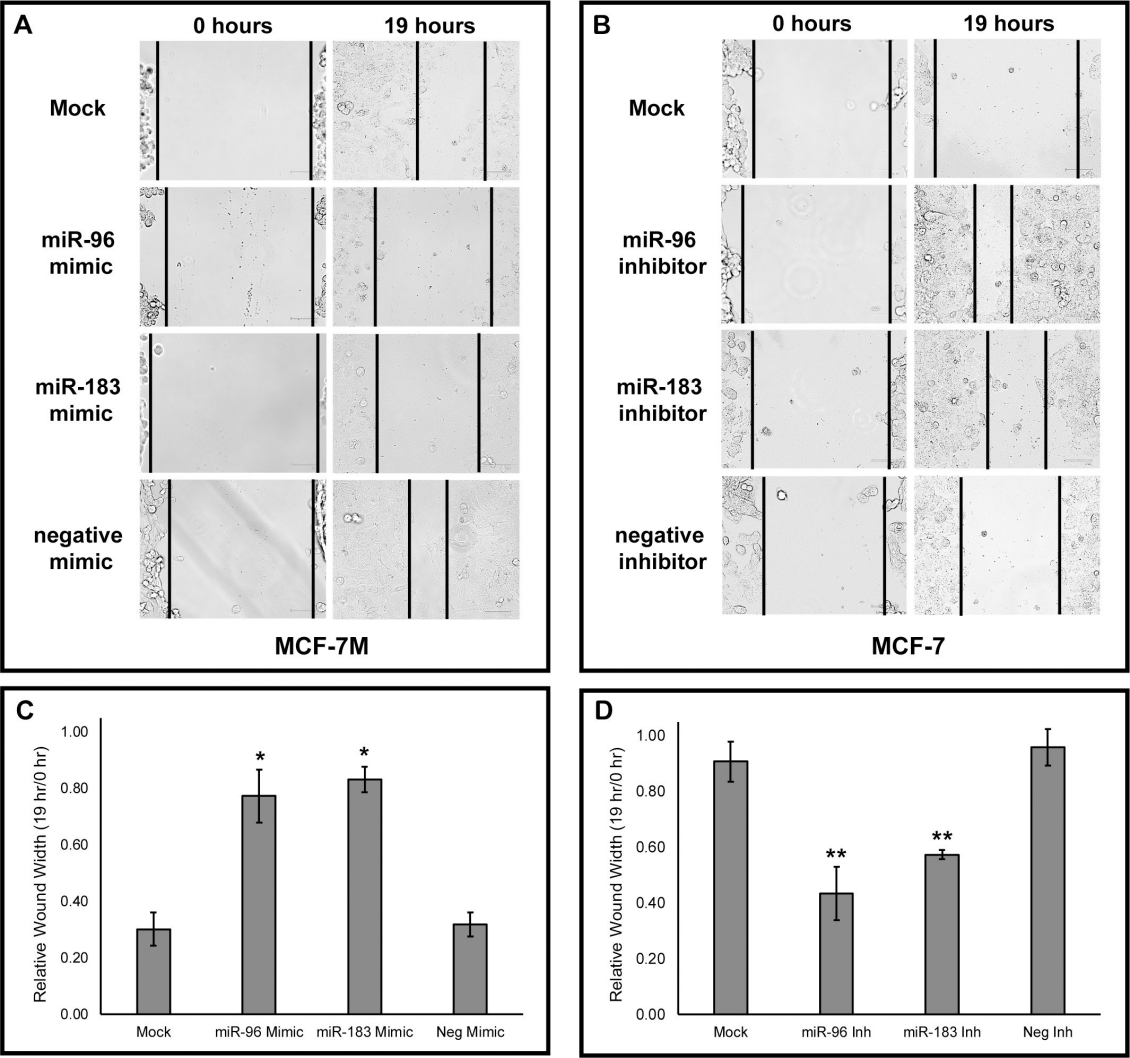
MicroRNAs miR-96 and miR-183 regulate cell migration. (A) 50 nM of mirVana^™^ miRNA mimics for miR-96, miR-183, or Negative Control #1 were transfected into MCF-7_M_ cells and (B) 50 nM of mirVana^™^ miRNA inhibitors for miR-96, miR-183, or Negative Control #1 were transfected into MCF-7 cells. Cells were allowed to grow for 24 hours post-transfection before each well was scraped in triplicate with a p-10 pipette tip. Images of wounds were taken using the EVOS FLoid Cell Imaging Station and are representative of three independent transfections. (C, D) Relative wound width was determined using ImageJ software (length of wound at 19 hours divided by length of wound at 0 hours) for MCF-7_M_ cells treated with miRNA mimics and MCF-7 cells treated with miRNA inhibitors (Inh). Values expressed as averages ± SEM. *p < .05, **p < .01.

Previous studies have shown that the miR-183-96-182 cluster may be involved in regulating cell proliferation and migration in various cancer models [7]. In order to determine if the downregulation of miR-96 and miR-183 plays a role in migration in a pre- and post-EMT breast cancer model, MCF-7_M_ cells were transfected with miR-96 and miR-183 microRNA mimics. MCF-7_M_ cells transfected with the miR-96 and miR-183 mimics had reduced closure of the wound (Fig 2A and 2C). Conversely, MCF-7 cells transfected with miR-96 and miR-183 inhibitors showed increased closure of the wound, suggesting an increased rate of migration (Fig 2B and 2D).

**Fig 3:**
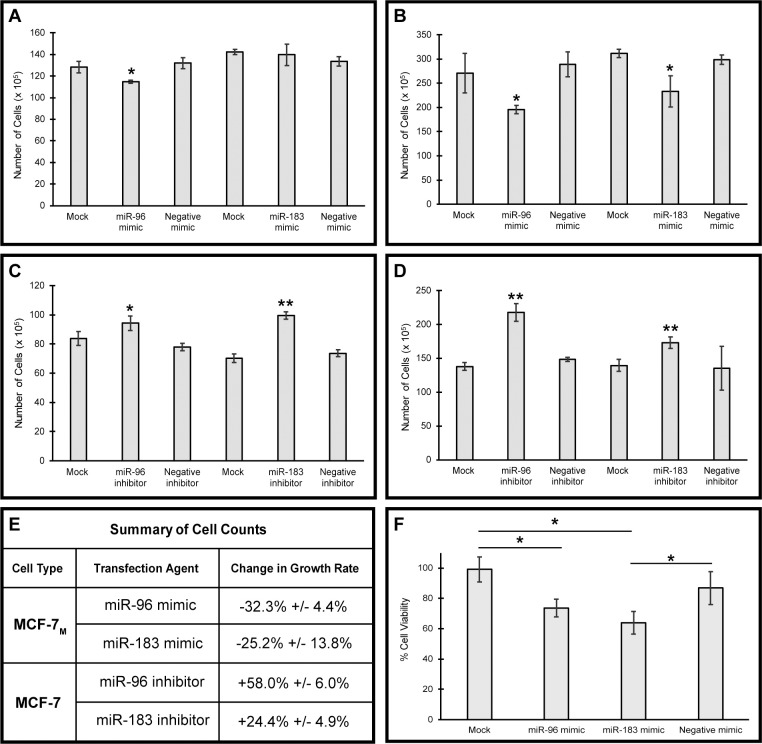
MicroRNAs miR-96 and miR-183 regulate cell viability. 50nM of mirVana^™^ miRNA mimics for miR-96, miR-183, or Negative Control #1 were transfected into MCF-7_M_ cells (A, B) and 50nM of mirVana^™^ miRNA inhibitors for miR-96, miR-183, or Negative Control #1 were transfected into MCF-7 cells (C, D). Cell counts were determined by staining with 0.4% trypan blue solution after 24 hours (A, C) or 48 hours (B, D), and averages and standard deviations of triplicate counts over three independent experiments are reported. (E) Calculated average percent change in viable cells for mock treatment vs. miRNA mimic or inhibitor treatment 48 hours post-transfection ± standard deviation. (F) A MTT Assay was performed to obtain an additional quantitative measure of cell viability in MCF-7_M_ cells. Statistical significance was determined using a one-way ANOVA followed by a Tukey-Kramer post-hoc test (*p < .05; **p < .01).

Additionally, a trypan blue cell viability assay was performed to better characterize the effects of miR-96 and miR-183 on cell growth. Increasing the expression of miR-96 and miR-183 in MCF-7_M_ cells via transfection with microRNA mimics resulted in significantly decreased cell viability, and this effect was more pronounced at 48 hours vs. 24 hours (Fig 3B and 3A, respectively). In contrast, decreasing the expression of miR-96 and miR-183 in MCF-7 cells via transfection with microRNA inhibitors resulted in a significant increase in cell viability, suggesting that the rate of cell proliferation may be increased following the inhibition of these microRNAs (Fig 3C and 3D). To confirm that the decreased number of cells observed in MCF-7_M_ cells treated with miR-96 and miR-183 mimics was due to reduced cell viability, a MTT assay was performed. Following transfection with miR-96 and miR-183 mimics, a statistically significant decrease in cell viability was observed as compared to both the mock and negative mimic samples (Fig 3F).

**Fig 4:**
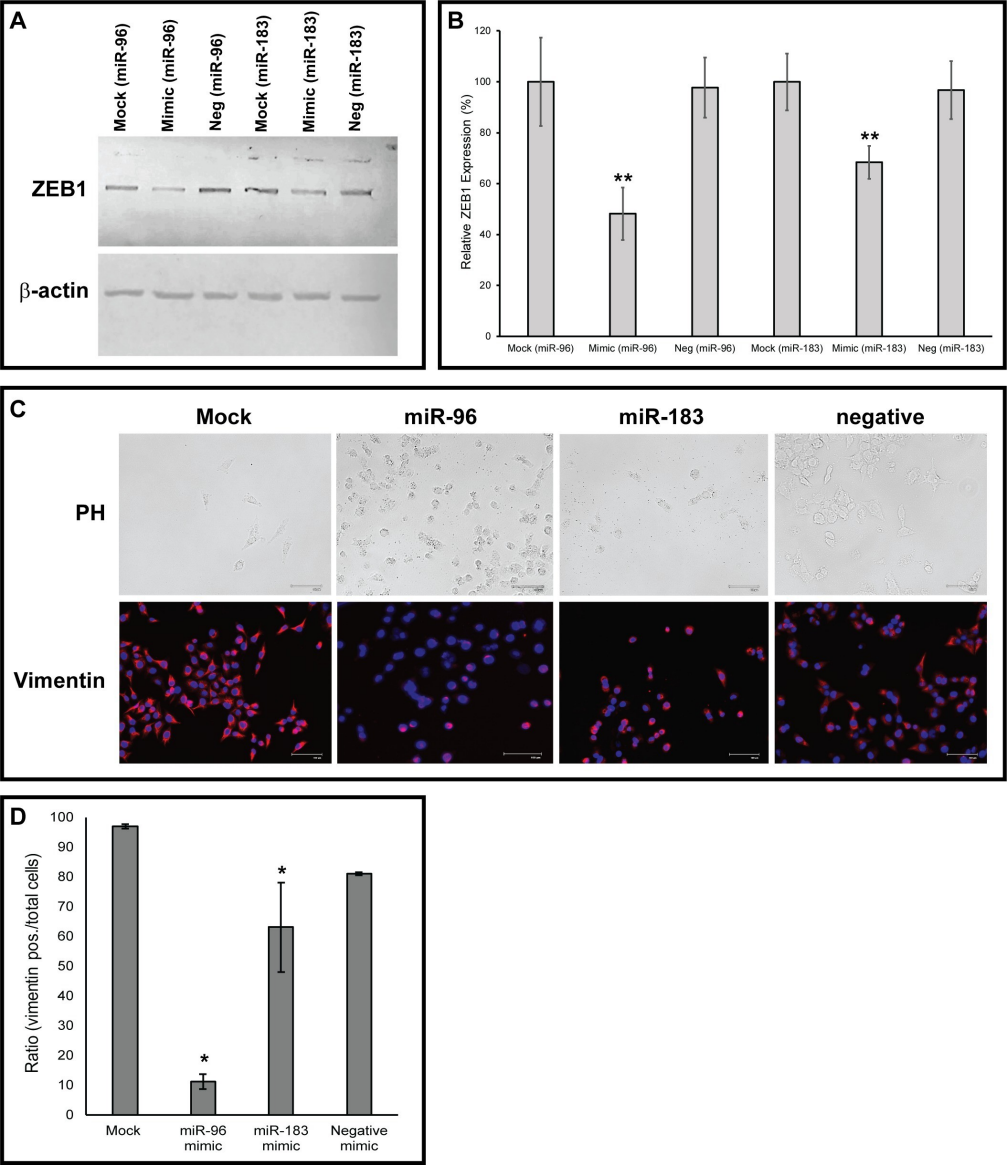
MicroRNAs miR-96 and miR-183 may regulate cell growth and migration via ZEB1. (A) Western blot of ZEB1 after the transfection of mirVana^™^ miRNA miR-96 and miR-183 mimics into MCF-7_M_ cells. β-actin was used as a loading control. (B) Quantitation of relative ZEB1 expression was measured by western blot analysis. Relative densities of ZEB1 and β-actin bands from two independent transfections were determined using ImageJ software. Values are expressed as average relative net ratios normalized to mock treatments ± standard deviation. (C) Immunofluorescence of vimentin expression in MCF-7_M_ cells following transfection with miR-96 and miR-183 mirVana^™^ miRNA mimics (bottom panel). In addition to decreased vimentin expression, cells developed an epithelial morphology following increased levels of miR-96 and miR-183 (top panel, PH = phase contrast). Scale bar = 100 μm. (D) Quantitation of immunofluorescence images from three independent experiments. Values expressed as the ratio of vimentin positive cells over total DAPI positive cells (average ± SEM). Statistical significance was determined using a one-way ANOVA followed by a Tukey-Kramer post-hoc test (*p, .05, **p < .01).

Previous studies have shown that the miR-183-96-182 cluster represses ZEB1, which in turn can downregulate transcription of this microRNA cluster in a reciprocal feedback loop [8]. In order to determine if this regulatory relationship was present in our model, MCF- 7_M_ cells were transfected with miR-96 and miR-183 mimics and the expression of ZEB1 protein was measured using western blot (Fig 4A). Following increased expression of miR-96 and miR-183, levels of ZEB1 were significantly decreased by 52% (miR-96) and 32% (miR-183) in comparison to mock and negative mimics treatments (Fig 4B). Immunofluorescence was then used to examine vimentin, a known marker of EMT and the mesenchymal phenotype. Vimentin was downregulated in MCF- 7_M_ cells treated with miR-96 and miR-183 mimics (Fig 4C and 4D). Significant differences were observed in levels of vimentin staining in MCF-7_M_ cells treated with miR-96 and miR-183 mimics compared to mock treatments. Levels of vimentin were also significantly lower in MCF-7_M_ cells treated with miR-96 mimics as compared to negative control mimics (Fig 4D). Though differences were seen in the level of vimentin staining in cells treated with miR-183 mimics compared to negative control mimics, this difference was not statistically significant, most likely due to a large standard deviation (Fig 4D). Furthermore, a more epithelial cuboidal morphology was observed in the MCF- 7_M_ cells treated with miR-96 and miR-183 mimics (Fig 4C), suggesting that the loss of these microRNAs may drive or maintain the mesenchymal phenotype during EMT.

## Discussion

The goal of this study was to further characterize the role of miR-96 and miR-183 in EMT in breast cancer. Previous studies have implicated these microRNAs in regulating cell proliferation and migration, however whether these microRNAs promote or inhibit these processes is unclear and seems to be context dependent [7]. Therefore, we used a pre- and post-EMT breast cancer cell culture model [6] to determine the role of the miR-183-96-182 cluster in regulating cell growth and migration. In our cell culture model, miR-96 and miR-183 (but not miR-182) were found to be downregulated in the post-EMT MCF-7_M_ cells (Fig 1). Due to this, it was hypothesized that loss of these microRNAs may facilitate EMT or aid in maintaining the mesenchymal phenotype.

To better characterize the role of miR-96 and miR-183 in the post-EMT mesenchymal MCF-7_M_ cells, the levels of these microRNAs were increased via transfection. This led to a significant reduction in cell viability and migration (Figs 2 and 3). This suggests that the loss of miR-96 and miR-183 during EMT may promote the more aggressive mesenchymal phenotype found in post-EMT cells. Additionally, when the levels of these microRNAs were decreased in the pre-EMT epithelial MCF-7 cells, the rate of cell growth and migration increased, further supporting that loss of these microRNAs promotes characteristics of EMT. To better understand the impact of miR-96 and miR-183 inhibition in MCF-7 cells, a BrdU cell proliferation assay could be performed to confirm if there is a bona fide increase in cell proliferation vs. an alternative mechanism driving the increase in cell viability.

Previous studies have shown that ZEB1 is a target of both miR-96 and miR-183 [8, 9]. To confirm this relationship in our breast cancer model of EMT, MCF-7_M_ cells were transfected with miR-96 and miR-183 mimics. Increasing the levels of these microRNAs in MCF-7_M_ cells resulted in decreased levels of ZEB1 protein (Fig 4). ZEB1 has been shown to repress E-cadherin, a protein that helps maintain an epithelial phenotype by promoting cell-to-cell adhesion [12]. Therefore, it is hypothesized that loss of E-cadherin contributes to the induction of EMT. We have previously shown that ZEB1 is increased in MCF-7_M_ vs. MCF-7 cells [6], which suggests that the loss of miR-96 and miR-183 during EMT may contribute to the increased levels of ZEB1 in MCF-7_M_ cells. Since EMT is reversible, repression of ZEB1 by increasing levels of miR-96 and miR-183 may also explain the observed decrease in cell viability and migration.

Additionally, MCF-7_M_ cells treated with miR-96 and miR-183 mimics showed a significant decrease in the expression of vimentin (Fig 4). Vimentin is a characteristic mesenchymal marker that is linked to a more aggressive phenotype [3]. This indicates that the loss miR-96 and miR-183 may play a role in maintaining the mesenchymal phenotype. This is further supported by the change in MCF-7_M_ cell morphology following treatment with miR-96 and miR-183 mimics. These cells displayed a marked loss of spindle shape, and a more rounded epithelial phenotype post-transfection (Fig 4). Though completely reverting mesenchymal cells back to an epithelial phenotype would likely involve coordination of a multitude of factors [8, 13], the reintroduction of miR-96 and miR-183 to MCF-7_M_ cells had statistically significant impacts on cell viability and migration. A study by Li et al. found that when isogenic colorectal cancer cells were transfected with miR-96 and miR-183 mimics, ZEB1 and vimentin were repressed, and there was a marked reduction in migration and invasion [8]. This supports the results of our study, indicating that miR-96 and miR-183 play a role in EMT, primarily through regulation of factors such as ZEB1 and vimentin.

Current research on microRNAs has generally focused on their use as a diagnostic tool [14, 15]. In regards to miR-96 and miR-183, a correlative relationship between these microRNAs and patient prognosis has been observed, indicating their potential use as biomarkers [16]. These microRNAs have also been linked to cell proliferation and migration in a multitude of cancers, including breast cancer [7]. These connections were further elucidated by the results of this study, confirming that miR-96 and miR-183 play a role of regulating cell growth and migration in a breast cancer model of EMT. By changing the levels of the microRNAs in pre- vs. post-EMT breast cancer cells (MCF-7 vs. MCF-7_M_), we confirmed that cell viability and migration are regulated in a reciprocal fashion by these microRNAs, and overexpression of miR-96 and miR-183 may reverse the mesenchymal phenotype observed in MCF-7_M_ cells.

While this study was able to further elucidate the role of miR-96 and miR-183 during EMT, these relationships are limited to our specific breast cancer cell model. While this does provide strength to the analysis by comparing cells of similar genetic backgrounds, these results should be confirmed in other models of EMT. Correspondingly, the consequence of manipulating these microRNAs in healthy cells is unknown.

In regards to clinical applications of microRNAs, there is the potential for off-target side-effects due to the fact that individual microRNAS could have hundreds of targets [17]. Therefore, while the loss of miR-96 and miR-183 may influence proteins in the EMT pathway, other cellular proteins may also be impacted, making it difficult to determine the complex effects of these microRNAs on all targets. Other studies have investigated the use of novel devices like microRNA mowers to change the levels of microRNAs in bladder cancer in order to reduce cell migration, proliferation, and induce apoptosis [18]. Moreover, there are many other microRNA-based therapeutics currently being developed to treat cancers, with some in clinical trials [19, 20]. These treatments are relatively non-invasive, ranging from intravenous to intratumoral injections to increase specificity and decrease side effects [19]. These therapeutic methodologies could be applied to miR-96 and miR-183 in post-EMT (i.e. metastatic) breast cancer cells in order to reduce the aggressive nature of these cells and decrease cell viability.

## Conclusions

Overall, this study demonstrates the role of miR-96 and miR-183 in regulating cell growth and migration in a breast cancer model of EMT. Additionally, miR-96 and miR-183 may play a role in maintaining a mesenchymal phenotype. This suggests that manipulating the levels of these microRNAs could have therapeutic applications. Therefore, in addition to helping slow down tumor growth and spread, miR-96 and miR-183 could also be used to reduce the size of tumors which could result in more diverse treatment options for patients. However, this study is limited to a breast cancer cell model, and future research should focus on the effects of these microRNAs systemically to determine their efficacy as a treatment for patients with breast cancer.

## Supporting information

S1 Table Primer sequences used for microRNA cDNA synthesis and PCR. (DOCX)

S1 Raw images Original images of PCR electrophoresis gels and western blots. (PDF)

S1 Data Human cancer RT2 microRNA PCR array complete data set. (XLSX)

S2 Data ImageJ analysis of microRNA gels. (XLSX)

S3 Data ImageJ analysis of wound healing images. (XLSX)

S4 Data Cell viability assay cell counts. (XLSX)

S5 Data MTT assay absorbance values. (XLSX)

S6 Data ImageJ analysis of ZEB1 western blots. (XLSX)

S7 Data Quantitation of immunofluorescence data. (XLSX)

S8 Data Wound healing images following transfection with miRNA mimics or inhibitors. (PDF)

S9 Data Immunofluorescence images of vimentin expression in MCF-7_M_ cells. (PDF)
