# Supplementary material for: Regulation of cell growth and migration by miR-96 and miR-183 in a breast cancer model of epithelial-mesenchymal transition
Source: PLoS One. 2020 May 12;15(5):e0233187. doi: 10.1371/journal.pone.0233187 (PMC7217431; doi:10.1371/journal.pone.0233187)
Supplement: S1 Table — (DOCX) [file pone.0233187.s001.docx]

|  | **Stem Loop Rt primer sequence** | **forward pcr primer** | **reverse pcr primer** |
| --- | --- | --- | --- |
| **miR-96** | 5’ GTCGTATCCAGTGCAGGGTCCGAGGTATTCGCACTGGATACGACAGCAAA 3’ | 5’ CGGCGGTTTGGCACTAGCACATT 3’ | 5’ CCAGTGCAGGGTCCGAGGTAT 3’ |
| **miR-182** | 5’ GTCGTATCCAGTGCAGGGTCCGAGGTATTCGCACTGGATACGACAGTGTG 3’ | 5’ CGGCGGTTTGGCAATGGTAGAACT 3’ | 5’ CCAGTGCAGGGTCCGAGGTAT 3’ |
| **miR-183** | 5’ GTCGTATCCAGTGCAGGGTCCGAGGTATTCGCACTGGATACGACagtgaa 3’ | 5’ CGGCGGTATGGCACTGGTAGAA 3’ | 5’ CCAGTGCAGGGTCCGAGGTAT 3’ |
| **miR-100** | 5’ GTCGTATCCAGTGCAGGGTCCGAGGTATTCGCACTGGATACGACcacaag 3’ | 5’ GGCGGaacccgtagatccgaa 3’ | 5’ CCAGTGCAGGGTCCGAGGTAT 3’ |
| **miR-200c** | 5’ GTCGTATCCAGTGCAGGGTCCGAGGTATTCGCACTGGATACGACtccatc 3’ | 5’ GGGGTAATACTGCCGGGTAAT 3’ | 5’ CCAGTGCAGGGTCCGAGGTAT 3’ |

**S1 Table. Primer Sequences used for microRNA cDNA synthesis and PCR**
