# Supplementary material for: Regulation of cell growth and migration by miR-96 and miR-183 in a breast cancer model of epithelial-mesenchymal transition
Source: PLoS One. 2020 May 12;15(5):e0233187. doi: 10.1371/journal.pone.0233187 (PMC7217431; doi:10.1371/journal.pone.0233187)
Supplement: S1 Raw images — (PDF) [file pone.0233187.s002.pdf]

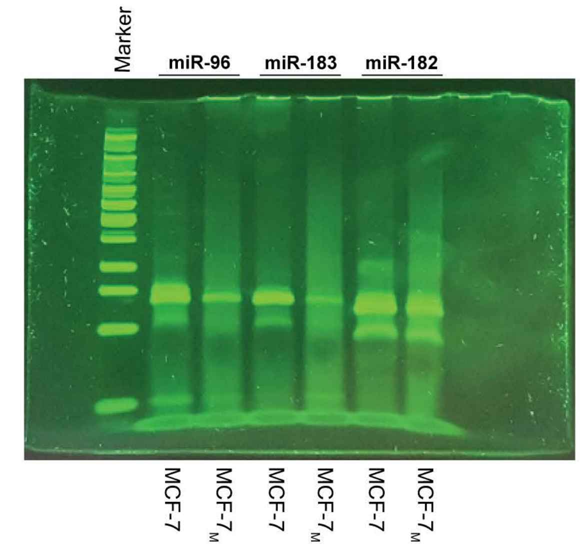

Raw Image 1. Relative expression levels of miR-96, miR-183, and miR-182 determined by PCR. Marker used was New England Biolabs Low Molecular Weight DNA Ladder. Expected product size = 72 bp. PCR products were resolved on a 10% non-denaturing acrylamide gel and stained with Sybr Gold. Image (grayscale) was presented in Fig 1B and used in quantitation of microRNA expression (Fig 1D).

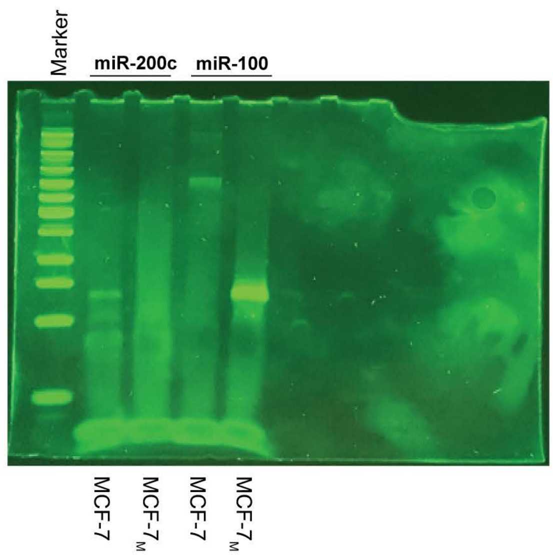

Raw Image 2. Relative expression levels of miR-200c and miR-100 determined by PCR. Marker used was New England Biolabs Low Molecular Weight DNA Ladder. Expected product size = 72 bp. PCR products were resolved on a 10% non-denaturing acrylamide gel and stained with Sybr Gold. Image (grayscale) was presented in Fig 1B and used in quantitation of microRNA expression (Fig 1D). .

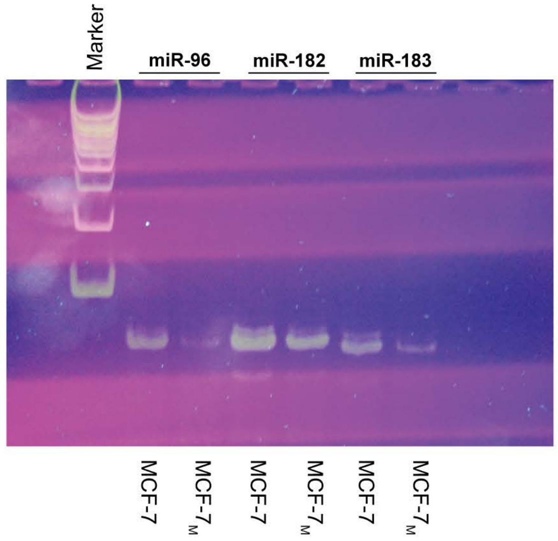

Raw Image 3. Relative expression levels of miR-96, miR-183, and miR-182 determined by PCR. Marker used was New England Biolabs 100bp DNA Ladder. PCR products stained with Sybr Gold and resolved on a 10% non-denaturing acrylamide gel. Image used in quantitation of microRNA expression (Fig 1D).

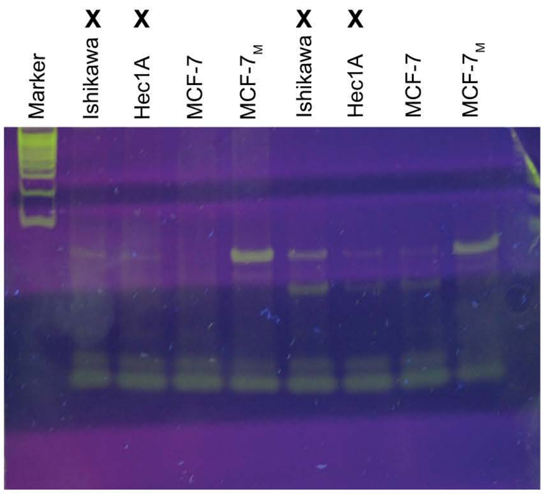

Raw Image 4. Relative expression levels of miR-100 in various cell lines determined by PCR. Each lane represents independent RNA/cDNA samples. Marker used was New England Biolabs 100bp DNA Ladder. Expected product size = 72 bp. PCR products stained with Sybr Gold and resolved on a 10% non-denaturing acrylamide gel. Image used in quantitation of microRNA expression (Fig 1D).

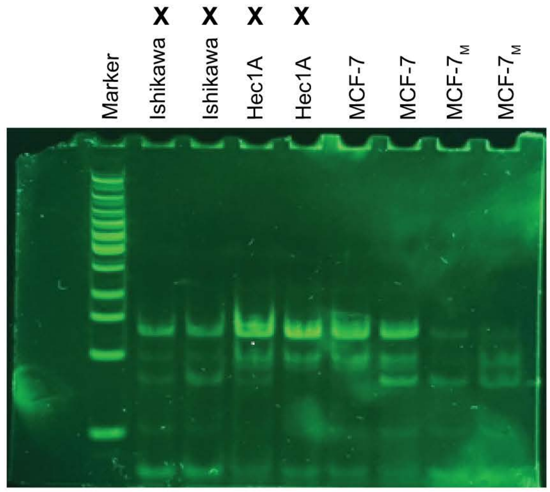

Raw Image 5. Relative expression levels of miR-200c in various cell lines determined by PCR. Each lane represents independent RNA/cDNA samples. Marker used was New England Biolabs Low Molecular Weight DNA Ladder. Expected product size = 72 bp. PCR products stained with Sybr Gold and resolved on a 10% non-denaturing acrylamide gel. Image used in quantitation of microRNA expression (Fig 1D).

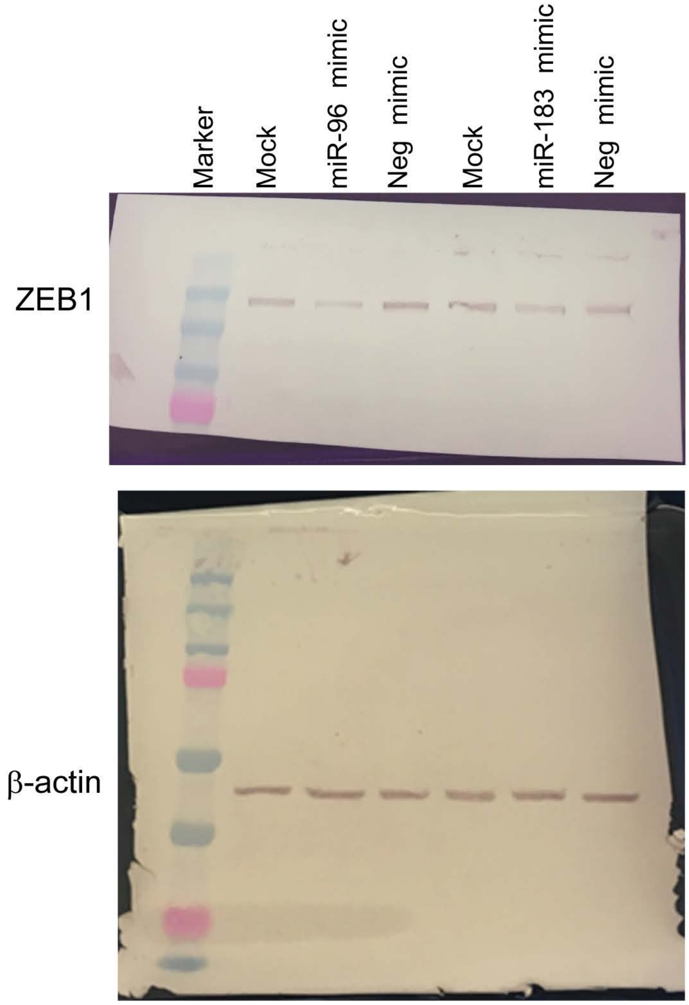

Raw Image 6. Western blot of ZEB1 expression in MCF-7M cells following transfection with miR-96 or miR-183 miRNA mimics, or a negative control miRNA mimic (Neg mimic). Each lane represents independent transfections. Proteins were detected using BCIP/NBT substrate following incubation with the species appropriate AP-conjugated secondary antibodies. Image obtained using a BioRad ChemiDoc MP system. β-actin used as a loading control. Marler used was BioRad Precision Plus Protein Dual Color Standards. Image (grayscale) presented in Fig 4A and used in ImageJ quantitation of ZEB expression (Fig 4B).

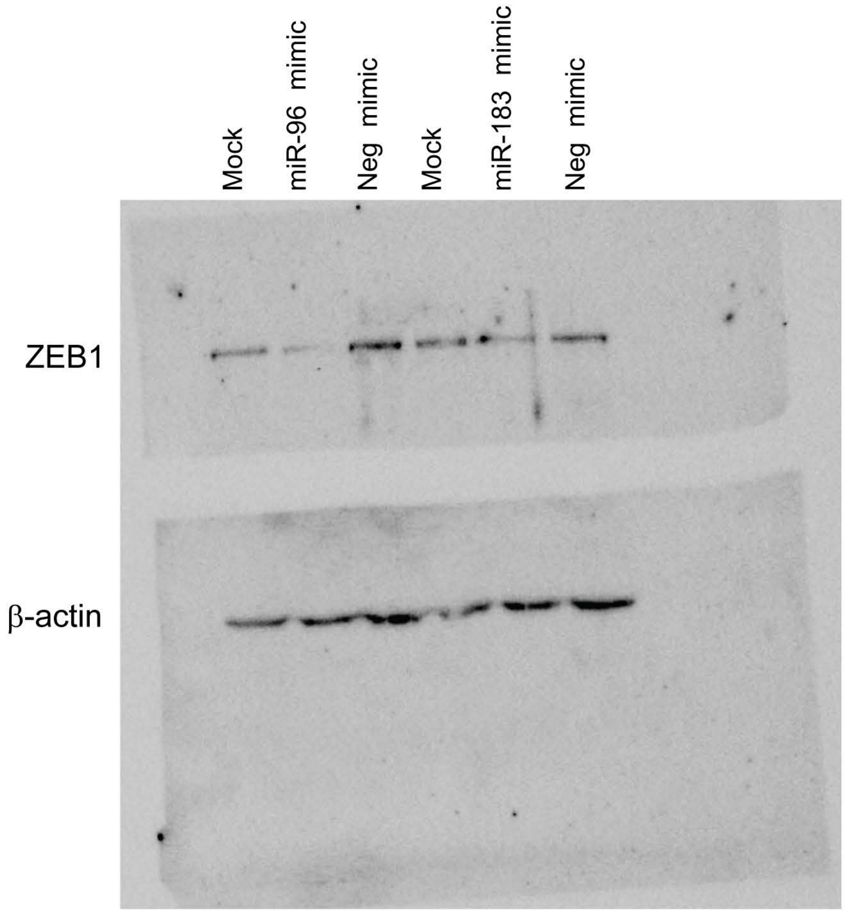

Raw Image 7. Western blot of ZEB1 expression in MCF-7M cells following transfection with miR-96 or miR-183 miRNA mimics, or a negative control miRNA mimic (Neg mimic). Each lane represents independent transfections. Proteins were detected using the BioRad Clarity ECL substrate following incubation with the species appropriate HRP-conjugated secondary antibodies. Image obtained using a BioRad ChemiDoc MP system. β-actin used as a loading control. Image used in ImageJ quantitation of western blot expression (Fig 4B).
