# Supplementary material for: Regulation of cell growth and migration by miR-96 and miR-183 in a breast cancer model of epithelial-mesenchymal transition
Source: PLoS One. 2020 May 12;15(5):e0233187. doi: 10.1371/journal.pone.0233187 (PMC7217431; doi:10.1371/journal.pone.0233187)
Supplement: S8 Data — (PDF) [file pone.0233187.s010.pdf]

MCF-7M – Time 0

Mock | miR-96 mimic | miR-183 mimic | Negative mimic

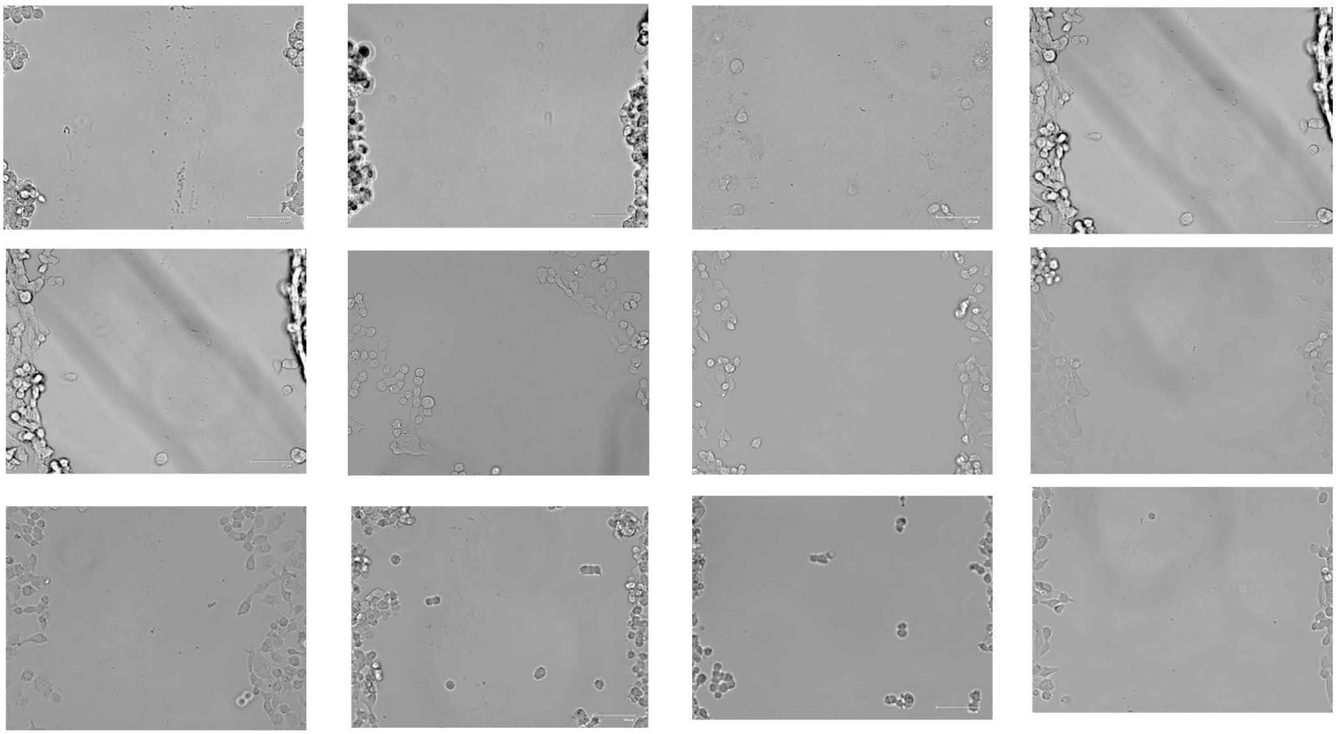

MCF-7M – Time 19 hr

Mock | miR-96 mimic | miR-183 mimic | Negative mimic

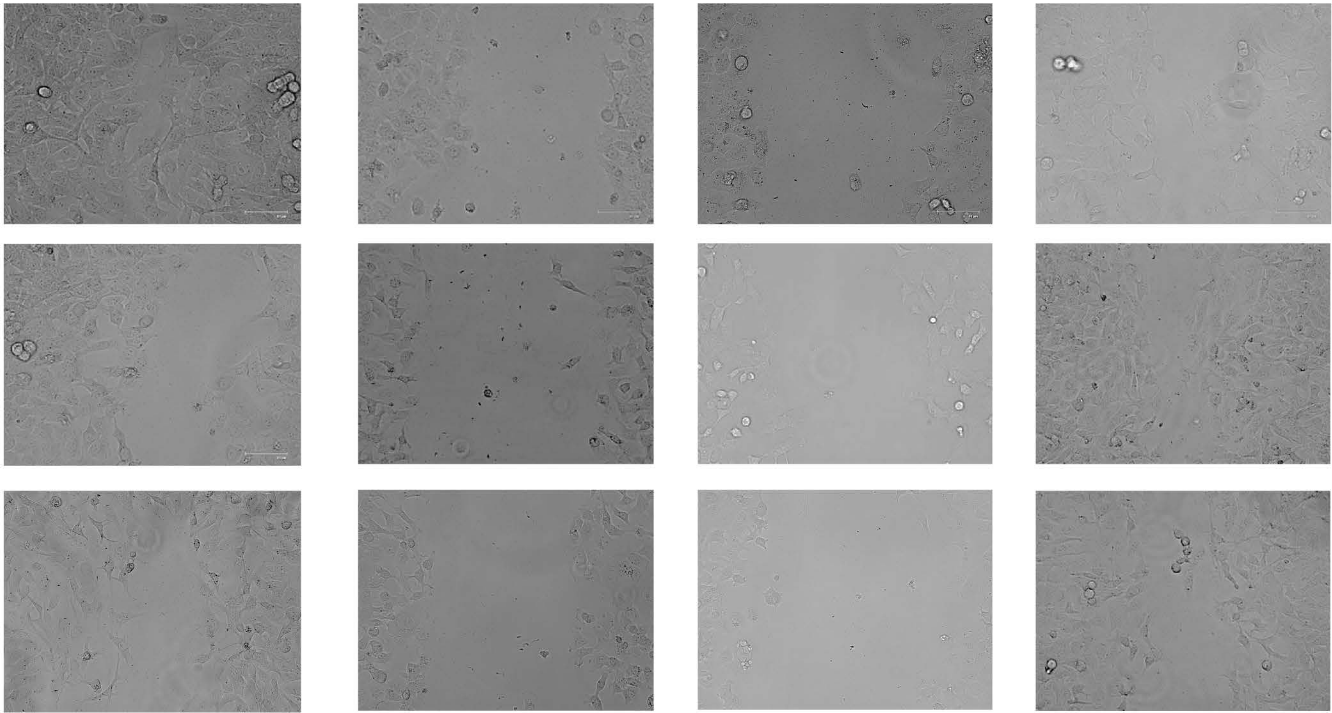

MCF-7 – Time 0

Mock | miR-96 inhibitor | miR-183 inhibitor | Negative inhibitor

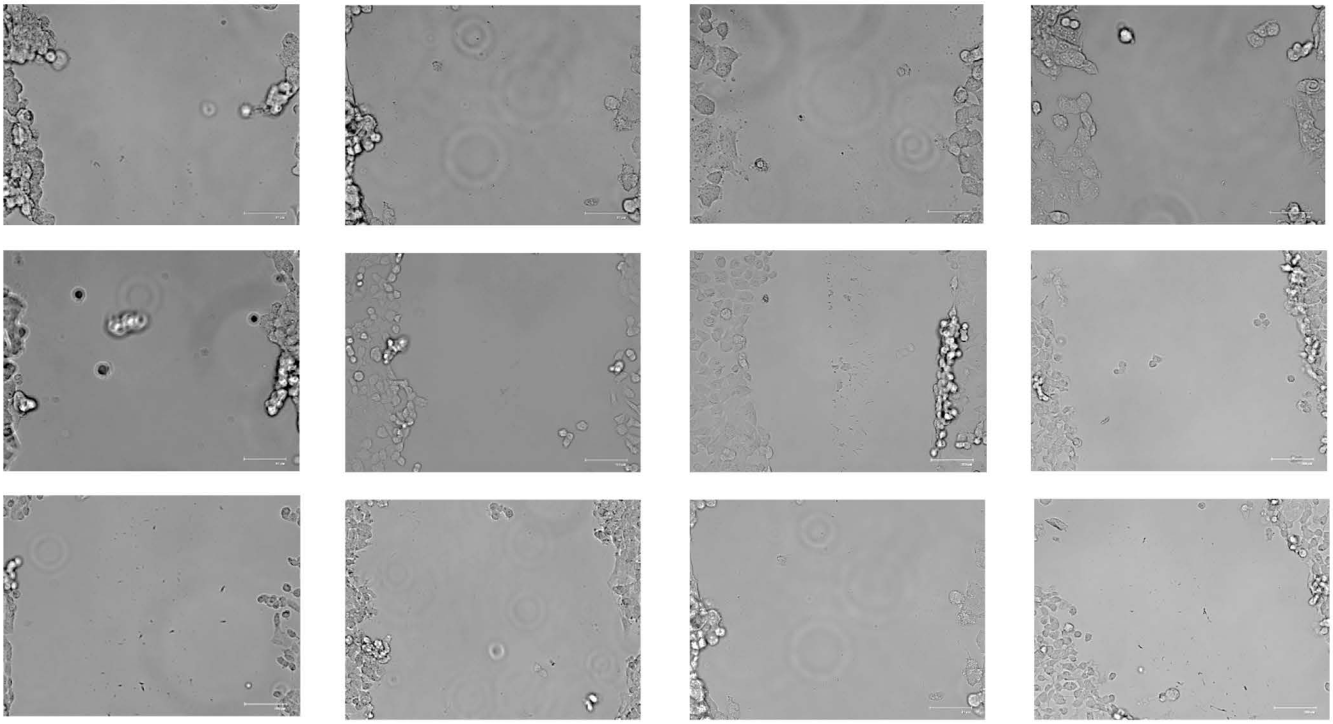

MCF-7 - Time 19 hr

Mock | miR-96 inhibitor | miR-183 inhibitor | Negative inhibitor

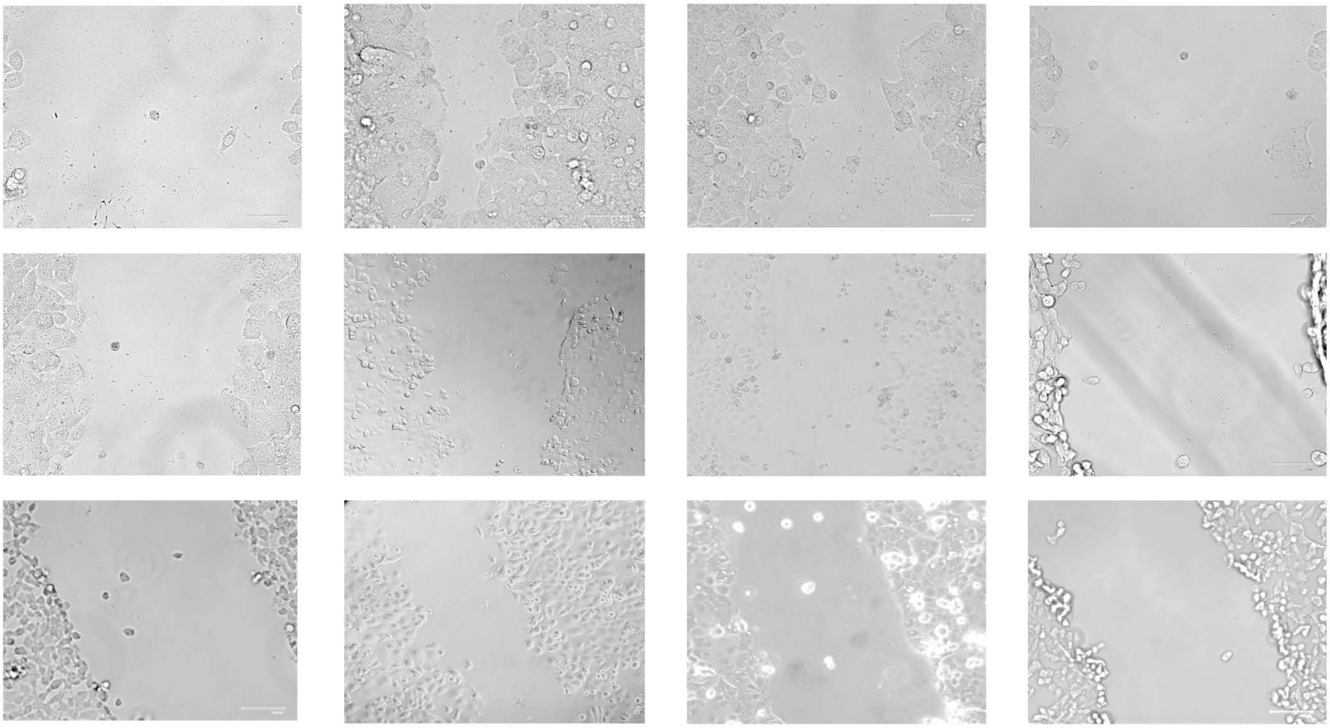

S8 Data. Wound healing images following transfection with miRNA mimics (MCF-7$_M$) or miRNA inhibitors (MCF-7). Images from three independent experiements (one experiment per row). Data used to quantitate relative wound width (length of wound at 19 hours divided by length of wound at 0 hours). Images presented in Figs 2A and 2B; quantitation presented in Figs 2C and 2D; statistical analysis in S3 Data.
