# Supplementary material for: Regulation of cell growth and migration by miR-96 and miR-183 in a breast cancer model of epithelial-mesenchymal transition
Source: PLoS One. 2020 May 12;15(5):e0233187. doi: 10.1371/journal.pone.0233187 (PMC7217431; doi:10.1371/journal.pone.0233187)
Supplement: S9 Data — (PDF) [file pone.0233187.s011.pdf]

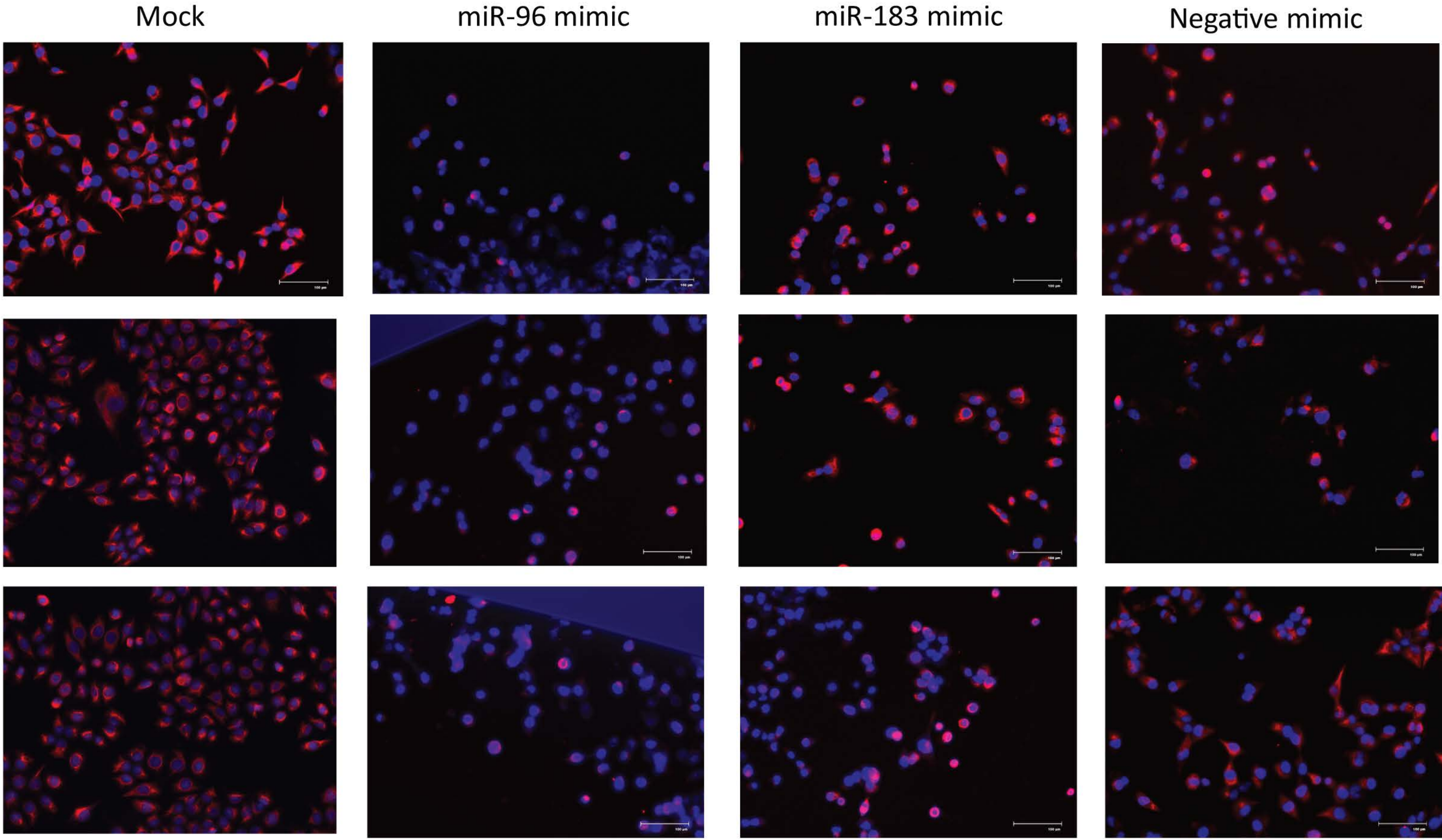

S9 Data. Immunofluorescence images of vimentin expression in MCF-7$_M$ cells. Images from three independent experiments (one experiment per row). Data used to quantitate relative vimentin expression in each field (number of vimentin positive cells divided by the total number of cells). Images presented in Fig 4C; quantitation presented in Fig 4D; statistical analysis in S7 Data.
